# Cardiovascular Risk Assessment in Psychotic Disorders: A Comparative Analysis of Plasma Atherogenic Index between Remitted Patients and Healthy Control

**DOI:** 10.1192/j.eurpsy.2024.599

**Published:** 2024-08-27

**Authors:** M. T. Ergün, B. Sağlıyan, Ö. Bayırlı, S. Kaya, M. Aydın

**Affiliations:** ^1^Department of Psychiatry, Selçuk University Faculty of Medicine Hospital, Konya, Türkiye

## Abstract

**Introduction:**

Psychiatric patients have a higher risk of premature mortality primarily due to cardiovascular diseases (CVD). One significant contributing factor is the presence of dyslipidemias . Current studies are shifting focus towards lipoprotein ratios, believed to better reflect cardiovascular risk. These studies have demonstrated that ratios associated with high-density lipoprotein (HDL) are stronger predictors for CVD compared to traditional lipid parameters. One of these ratios is the logarithmic transformation of the triglyceride (TG) to HDL ratio, known as the plasma atherogenic index (PAI).

**Objectives:**

Our study aimed to compare the PAI between patients diagnosed with psychotic disorders who presented to our outpatient clinic and healthy control groups.

**Methods:**

Fifty patients diagnosed with psychotic disorders, including 50 residing in a nursing home and 50 outpatient in such facilities, presented to our psychiatric outpatient clinic and were included in our study. Additionally, a healthy control group consisting of 49 individuals was recruited. A socio-demographic data form was administered to all groups. Peripheral blood levels of HDL, Triglycerides (TG), and LDL were recorded for each participant included in the study. Ethical approval for the study was obtained from the local ethics committee.

**Results:**

The patient groups were compared in terms of age and gender. While there was no statistically significant difference in gender between the groups, a significant difference was observed in terms of age (p=0.099, p=0.004). When examining the age distribution of the groups, it was observed that the care facility group was older compared to the other groups. The age and gender distributions of the groups are shown in Table 1 and Table 2.

Psychotic patients in the outpatient group and the nursing home group were compared in terms of age and atherogenic index. Age was statistically significant, indicating that thenursing home group was significantly older (p=0.001, p=0.478). In the comparison of the control group with psychotic patients, there was no statistical difference in age, but a significant difference was found in terms of the atherogenic index (p=0.510, p=0.001). The statistical analysis and data between psychotic patients and the control group are presented in Table 3.

**Image:**

**Figure:**
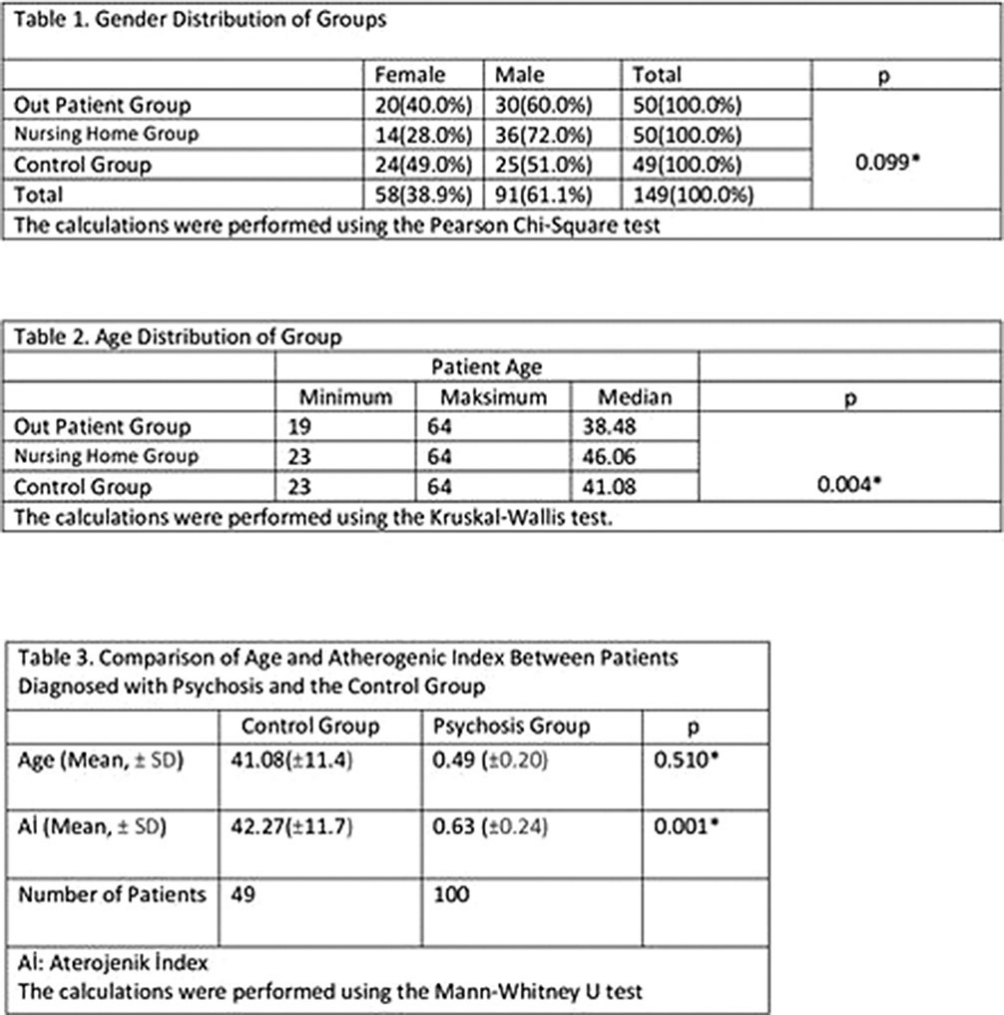

**Conclusions:**

This study, examining the comparison of Plasma Atherogenic Index (PAI) in patients diagnosed with psychosis with healthy controls, represents a significant step in understanding the cardiovascular health profile of this population and developing appropriate treatment strategies. Future research will further contribute to a deeper understanding of the impact of psychiatric disorders on cardiovascular health and aid in the development of effective interventions to minimize these effects.

Disclosure of Interest: None Declared.

**Disclosure of Interest:**

None Declared

